# Maternal early pregnancy dietary glycemic index and load, fetal growth, and the risk of adverse birth outcomes

**DOI:** 10.1007/s00394-020-02327-9

**Published:** 2020-07-14

**Authors:** Rama J. Wahab, Judith M. Scholing, Romy Gaillard

**Affiliations:** 1 The Generation R Study Group, Erasmus MC, University Medical Center, Rotterdam, The Netherlands; 2 Department of Pediatrics, Sophia’s Children’s Hospital, Erasmus MC, University Medical Center, Rotterdam, The Netherlands; 3 Division of Human Nutrition and Health, Wageningen University and Research, Wageningen, The Netherlands

**Keywords:** Glycemic index, Glycemia, Pregnancy, Fetal growth, Birth outcomes

## Abstract

**Purpose:**

Maternal hyperglycemia is associated with adverse birth outcomes. Maternal dietary glycemic index and load influence postprandial glucose concentrations. We examined the associations of maternal early pregnancy dietary glycemic index and load with fetal growth and risks of adverse birth outcomes.

**Methods:**

In a population-based cohort study of 3471 pregnant Dutch women, we assessed dietary glycemic index and load using a food frequency questionnaire at median 13.4 (95% range 10.6; 21.2) weeks gestation. We measured fetal growth in mid- and late-pregnancy by ultrasound and obtained birth outcomes from medical records.

**Results:**

Mean maternal early pregnancy dietary glycemic index and load were 57.7 (SD 3.3, 95% range 52.8; 63.5) and 155 (SD 47, 95% range 87; 243), respectively. Maternal early pregnancy dietary glycemic index was not associated with fetal growth parameters. A higher maternal early pregnancy dietary glycemic load was associated with a higher fetal abdominal circumference and estimated fetal weight in late-pregnancy (*p* values < 0.05), but not with mid-pregnancy or birth growth characteristics. A higher maternal early pregnancy dietary glycemic index was associated with a lower risk of a large-for-gestational-age infant (*p* value < 0.05). Maternal early pregnancy glycemic index and load were not associated with other adverse birth outcomes.

**Conclusion:**

Among pregnant women without an impaired glucose metabolism, a higher early pregnancy dietary glycemic load was associated with higher late-pregnancy fetal abdominal circumference and estimated fetal weight. No consistent associations of maternal dietary glycemic index and load with growth parameters in mid-pregnancy and at birth were present. A higher glycemic index was associated with a lower risk of a large-for-gestational-age infant.

**Electronic supplementary material:**

The online version of this article (10.1007/s00394-020-02327-9) contains supplementary material, which is available to authorized users.

## Introduction

Maternal hyperglycemia during pregnancy is a well-known risk factor for adverse birth outcomes, such as macro-somia and neonatal hypoglycemia [1]. Accumulating evidence suggests that early pregnancy is a critical period for the adverse effects of high maternal glucose concentrations on embryonic and placental development [2, 3]. High maternal glucose concentrations from early pregnancy onwards may cause alterations in embryonic and placental development, and lead to an increased transfer of glucose to the developing fetus, predisposing to increased fetal growth and fat deposition and alterations in fetal metabolism. These fetal adaptations may, subsequently, predispose to increased risks of adverse birth outcomes [1].

During pregnancy, most transfer of glucose across the placenta occurs in the postprandial state. These postprandial glucose concentrations are mainly determined by maternal dietary carbohydrate intake [4]. The dietary glycemic index and glycemic load are measures that can be used to qualify and quantify the maternal postprandial glycemic response to the maternal dietary carbohydrate intake. These measures influence postprandial glucose available for maternal energy, storage, and transfer to the fetus [5, 6]. Intervention studies suggested that a low-glycemic index diet during the second half of pregnancy may reduce birth weight and infant adiposity in women with gestational diabetes or an impaired glucose metabolism [7, 8]. No increased risks of delivering a small-for-gestational-age infant were observed in these intervention studies. However, an observational study among pregnant women not at risk of an impaired glucose metabolism reported that a lower.

Maternal dietary glycemic index in the second half of pregnancy was associated with an increased risk of delivering a small-for-gestational-age infant [9]. In pregnant women without an impaired glucose metabolism, not much is known about the effects of maternal dietary glycemic index and load during early pregnancy on directly measured fetal growth throughout pregnancy and the risks of adverse birth outcomes. We hypothesized that a lower maternal dietary glycemic index and load in early pregnancy might reduce the risks of fetal overgrowth and macro-somia, but might also lead to increased risks of fetal undergrowth and low birth weight, especially among a general, healthy population.

Therefore, in a population-based prospective cohort study among 3471 pregnant women without an impaired glucose metabolism, we examined the associations of maternal early pregnancy dietary glycemic index and load within a low-to-normal range with fetal growth throughout pregnancy and the risks of adverse birth outcomes.

## Methods

### Study design and study sample

This study was embedded in the Generation R study, a population-based prospective birth cohort study in Rotterdam, The Netherlands. Details of the study have been described previously [10]. Written informed consent was obtained from all women at enrollment between April 2002 and January 2006. The response rate at baseline was 61%, which was calculated by dividing the number of participating live born children by the total number of live born children born in the study area during the inclusion period. The study was approved by the Medical Ethical Committee of Erasmus MC University Medical Center in Rotterdam, The Netherlands (MEC 198.782/2001/31). In total, 4544 Dutch women were enrolled during pregnancy. During early pregnancy, information on dietary intake was available in 3558 Dutch women. After exclusion of women with pre-gestational diabetes and non-singleton live births, the final study sample consisted of 3471 pregnant women and their newborns.

### Maternal dietary glycemic index and load

We obtained information on maternal dietary intake during early pregnancy at a median of 12.9 weeks gestation (95% range 10.4; 16.8) by a semi-quantitative 293-item Food Frequency Questionnaire (FFQ) [11]. The FFQ was validated against three 24-h dietary recalls in 71 pregnant women with Dutch ethnicity living in Rotterdam. Intra-class correlation coefficients for macronutrient intakes ranged from 0.50 to 0.70 and were 0.54 for carbohydrate intake [12]. The average energy intake and carbohydrate intake was calculated using the Dutch Food Composition Table 2006 [13]. Next, we calculated the maternal early pregnancy dietary glycemic index and load. The dietary glycemic index provides information on the quality of the glycemic response to a carbohydrate containing food product and is more often used in intervention studies and clinical settings [14, 15]. The dietary glycemic load additionally takes the amount of carbohydrate intake into account and, therefore, provides additional information on maternal postprandial glucose concentrations, but this measure may be more prone to measurement errors [5, 6, 16]. In line with previous observational studies, we calculated both maternal dietary glycemic index and load for the current study [17–19]*.* To calculate maternal early pregnancy dietary glycemic index and load, glycemic index values were assigned to each individual food item in the FFQ. Glycemic index values were obtained from the glycemic index database on the Dutch diet published by the Medical Research Council Human Nutrition Research (MRC HNR), Cambridge, United Kingdom, using glucose as a reference (glycemic index for glucose equal to 100) [20]. Using this database, we obtained direct matches for 84.3% of the food items. For the food items that could not directly be matched in the database, glycemic index values for similar food items were obtained from proxies (87.8%) or from glycemic index databases of MRC HNR for other countries (9.8%). If no equivalent food item was available for a food item, an arbitrary value of 70 was assigned according to the procedure developed by the MRC HNR (2.4%) [18, 20].

The mean maternal dietary glycemic index per day was calculated by summing the product of the carbohydrate intake of each food item with its glycemic index, which was divided by the total amount of carbohydrates consumed per day. The mean maternal dietary glycemic load was calculated by summing the product of the carbohydrate intake of each food item with the glycemic index of that specific food item. We constructed quartiles and standard deviation scores of maternal dietary glycemic index and glycemic load.

Intervention studies stimulate a low-glycemic index diet by recommending an exchange of high-glycemic index products for low-glycemic index products, which results in a low mean dietary glycemic index [19, 21]. In line with these studies, we aimed to explore the effects of a low-glycemic index diet on fetal growth and birth characteristics and the risk of adverse birth outcomes as a secondary analysis. We categorized the mean maternal dietary glycemic index per day into a low, normal, and high-glycemic index diet, using similar cut-offs as used for individual food products [low-glycemic index diet (≤ 55), a normal-glycemic index diet (56–69), and a high-glycemic index diet (≥ 70)]. We consider this approach in line with intervention studies who recommend a low-glycemic index diet through eating low-glycemic index food products [19, 21].

### Fetal growth and adverse birth outcomes

We performed fetal ultrasound examinations to assess fetal growth during mid- and late-pregnancy at a median gestational age of 20.5 (95% range 19.0; 22.6) and 30.4 (95% range 28.9; 32.2) weeks, respectively. Gestational age was established during early pregnancy based on crown-rump length. During mid- and late-pregnancy, we measured femur length, abdominal circumference, and head circumference to the nearest millimeter using standardized ultrasound procedures [22]. Head circumference, abdominal circumference, and femur length were used to estimate fetal weight by using the Hadlock equation [23]. Longitudinal growth curves and gestational-age-adjusted standard deviation scores (SDS) were constructed for all fetal growth measurements [24]. These gestational-age-adjusted SDS were based on reference growth curves from the whole study population, and represent the equivalent of *z* scores.

We obtained data on gestational age, weight, length, and head circumference at birth from medical records. Because head circumference and length were not routinely measured at birth, fewer measurements were available (*n* = 1942 for head circumference and *n* = 2323 for length at birth). Gestational-age-adjusted SDS for birth weight, length, and head circumference were constructed using North European growth standards [25]. Based on international guidelines, we defined small-for-gestational-age and large-for-gestational-age at birth as the lowest and the highest ten percentiles of gestational-age-adjusted birth weight within our study population, respectively [19, 26]. Preterm birth was defined as a gestational age at birth < 37 weeks [25]. Information on caesarian delivery was obtained from medical records.

### Covariates

Information on maternal age, educational level (primary education finished, secondary education finished, and higher education finished), parity (nulliparous and multiparous), folic acid supplement use (yes/no), and daily nausea for past three months (yes/no) and daily vomiting for past 3 months (yes/no) was collected by questionnaire at enrollment. We measured maternal height at enrollment and obtained information on maternal pre-pregnancy weight through questionnaire and calculated pre-pregnancy body mass index (BMI) [10]. Information on maternal smoking (yes/no) and alcohol consumption (yes/no) was assessed by repeated questionnaires throughout pregnancy [10]. Information on gestational diabetes was obtained through medical records.

### Statistical analyses

First, we performed a non-response analysis comparing Dutch women with and without information available on early pregnancy dietary glycemic index and load. We further compared population characteristics according to maternal dietary glycemic index quartiles using Chi-square tests for categorical variables and one-way ANOVA for continuous variables. Second, we examined the associations of maternal early pregnancy dietary glycemic index and load with fetal growth patterns from mid-pregnancy onwards using unbalanced repeated measurement regression models. We included maternal early pregnancy dietary glycemic index and load quartiles in these models as intercept and as interaction term with gestational age to estimate fetal growth rates over time. To further assess the associations of maternal dietary glycemic index and load with fetal growth characteristics in each pregnancy period in detail, we examined the associations of maternal early pregnancy dietary glycemic index and load in quartiles and per SDS change with each fetal growth characteristics in each pregnancy period and at birth using linear regression models. In the analyses with maternal early pregnancy dietary glycemic index and load in quartiles, we assessed whether associations were restricted to women with a relatively low or high dietary glycemic index and load and explored whether there was a linear tendency present. We used quartiles based on variability between the categories of maternal dietary glycemic index and load and to maintain statistical power. Next, we assessed the associations of maternal early pregnancy dietary glycemic index and load continuously per 1-SDS increase with fetal growth characteristics in each pregnancy period and at birth to explore the continuous associations across the low-to-normal range of maternal early pregnancy dietary glycemic index and load, which is not fully captured by the quartile analyses. First, we only adjusted for gestational age at study enrollment. Subsequently, we additionally adjusted these models for maternal age, parity, educational level, pre-pregnancy BMI, early pregnancy total daily energy intake, smoking during pregnancy, alcohol use during pregnancy, daily nausea, and vomiting during early pregnancy and fetal sex, as nutritional exposures are prone to confounding by other maternal socio-demographic and lifestyle characteristics. Variables were selected based on literature and included in the final model when the covariate caused a ≥ 10% change in the effect estimate [27–29]. We did not adjust for gestational weight gain, as fetal growth is a major component of gestational weight gain and additional adjustment of gestational weight gain would thus lead to over adjustment. Finally, we assessed the associations of maternal early pregnancy dietary glycemic index and load in quartiles and per SDS change with the risks of adverse birth outcomes using logistic regression models with similar adjustment. To assess whether the effects were different for mothers with a different pre-pregnancy BMI and/or child’s sex, we tested for interactions between maternal dietary glycemic index and load and maternal pre-pregnancy BMI and child’s sex in the models described above, but none were significant [30, 31].

We performed several sensitivity analyses: (1) as a secondary analysis, we further explored the associations of a low-glycemic index diet as compared to a normal-glycemic index diet, according to our predefined categories, with fetal growth and the risks of adverse birth outcomes; (2) as we were interested in the effects of maternal dietary glycemic index and load among low-risk pregnant women, we repeated the analyses excluding women with gestational diabetes, excluding overweight and obese women and excluding women aged > 35 years, respectively [14, 32].

To reduce selection bias due to missing data, multiple imputations of covariates (pooled results of five imputed datasets) were be performed [33]. The repeated measurement analyses were performed using the Statistical Analysis System version 9.4 (SAS Institute, Cary, NC, USA). All other analyses were performed using the Statistical Package of Social Sciences version 24.0 for Windows (SPSS Inc., Chicago, IL, USA).

## Results

### Subject characteristics

Mean maternal dietary glycemic index and load were 57.7 (SD 3.3, 95% range 52.8; 63.5) and 155 (SD 47, 95% range 87; 243), respectively (Table 1). 705 (20.3%) women consumed a low-glycemic index diet (mean dietary glycemic index per day ≤ 55) and no women consumed a high-glycemic index diet (mean glycemic index per day ≥ 70). Women within the higher dietary glycemic index quartiles were more likely to be younger, multiparous, lower educated, had a higher pre-pregnancy BMI, higher total energy intake, and smoked more often during pregnancy. Fetal growth characteristics according to maternal dietary glycemic index quartiles are given in Supplementary Table S1**.** The non-response analysis showed that women with information on dietary intake were more likely to be multiparous and higher educated compared to women without these data (Supplementary Table S2).

**Table 1:** Population characteristics according to maternal dietary glycemic index quartiles

	Total group (*n* = 3471)	Glycemic index quartile 1 (*n* = 867)	Glycemic index quartile 2 (*n* = 868)	Glycemic index quartile 3 (*n* = 868)	Glycemic index quartile 4 (*n* = 868)	*p* value^a^
Maternal characteristics						
Maternal age at enrollment, mean (SD), years	31.4 (4.4)	32.3 (4.0)	31.7 (4.1)	31.1 (4.4)	30.4 (4.9)	0.00
Gestational age at enrollment, median (95%), weeks	13.4 (10.6; 21.2)	13.4 (10.9; 21.2)	13.4 (10.5; 21.6)	13.4 (10.6; 21.6)	13.4 (10.4; 20.8)	0.93
Parity, *n* nulliparous (%)	2076 (59.9)	542 (62.7)	538 (62.0)	513 (59.2)	483 (55.9)	0.02
Pre-pregnancy weight status, overweight, or obese, *n* (%)	685 (22.9)	138 (18.3)	164 (22.2)	194 (25.9)	189 (25.3)	0.01
Gestational weight gain, mean (SD), g/week	10.8 (4.4)	10.7 (4.1)	10.8 (4.5)	10.9 (4.4)	10.8 (4.7)	0.71
Education, *n* high (%)	2026 (59.1)	605 (70.4)	536 (62.3)	489 (57.3)	396 (46.4)	0.00
Glycemic index, mean (SD)	57.7 (3.3)	53.8 (1.4)	56.5 (0.6)	58.6 (0.7)	62.1 (1.9)	n.a.
Glycemic load, mean (SD)	155 (47)	132 (33)	147 (39)	160 (43)	179 (56)	0.00
Carbohydrate intake, mean (SD), g/day	267 (75)	246 (60)	261 (68)	272 (74)	288 (88)	0.00
Protein intake, mean (SD), g/day	79 (19)	82 (18)	80 (19)	79 (19)	75 (20)	0.00
Fat intake, mean (SD), g/day	86 (24)	85 (24)	87 (24)	88 (25)	85 (25)	0.02
Fiber intake, mean (SD), g/day	23 (7)	25 (7)	24 (7)	23 (7)	21 (7)	0.00
Total energy intake, mean (SD), kcal/day	2145 (511)	2063 (453)	2132 (495)	2183 (516)	2201 (564)	0.00
Folic acid supplement use, *n* yes (%)	2532 (72.9)	658 (75.9)	638 (73.5)	648 (74.7)	588 (67.7)	0.00
Alcohol use during pregnancy, *n* yes (%)	2117 (66.3)	581 (72.8)	554 (69.6)	506 (63.1)	476 (59.6)	0.00
Smoking during pregnancy, *n* yes (%)	833 (26.9)	152 (19.0)	181 (22.5)	226 (28.0)	274 (33.9)	0.00
Nausea during early pregnancy, *n* (%)	880 (27.7)	181 (22.8)	207 (26.2)	249 (31.2)	243 (30.5)	0.00
Vomiting during early pregnancy, *n* (%)	145 (4.6)	20 (2.5)	36 (4.6)	42 (5.3)	47 (5.9)	0.01
Gestational diabetes, *n* (%)	31 (0.9)	6 (0.7)	6 (0.7)	9 (1.1)	10 (1.2)	0.64
Birth characteristics						
Sex, *n* male (%)	1753 (50.5)	404 (46.6)	475 (54.7)	411 (47.4)	463 (53.3)	0.00
Gestational age at birth, median (95% range), weeks	40.3 (37.0, 42.1)	40.3 (36.9, 42.1)	40.3 (37.1, 42.1)	40.1 (37.1, 42.1)	40.3 (36.3, 42.1)	0.59
Birthweight, mean (SD), g	3489 (554)	3498 (584)	3500 (512)	3505 (553)	3452 (565)	0.17
Preterm birth, *n* (%)	162 (4.7)	43 (5.0)	34 (3.9)	30 (3.5)	55 (6.3)	0.02
Small-for-gestational-age, *n* (%)	345 (10.0)	85 (9.8)	82 (9.5)	87 (10.1)	91 (10.5)	0.91
Large-for-gestational-age, *n* (%)	345 (10.0)	105 (12.1)	85 (9.8)	89 (10.3)	66 (7.6)	0.02

^a^*p* values were obtained by ANOVA test for continuous variables or by Chi-square test for categorical variables

### Maternal dietary glycemic index and load and fetal growth

**Fig. 1:**
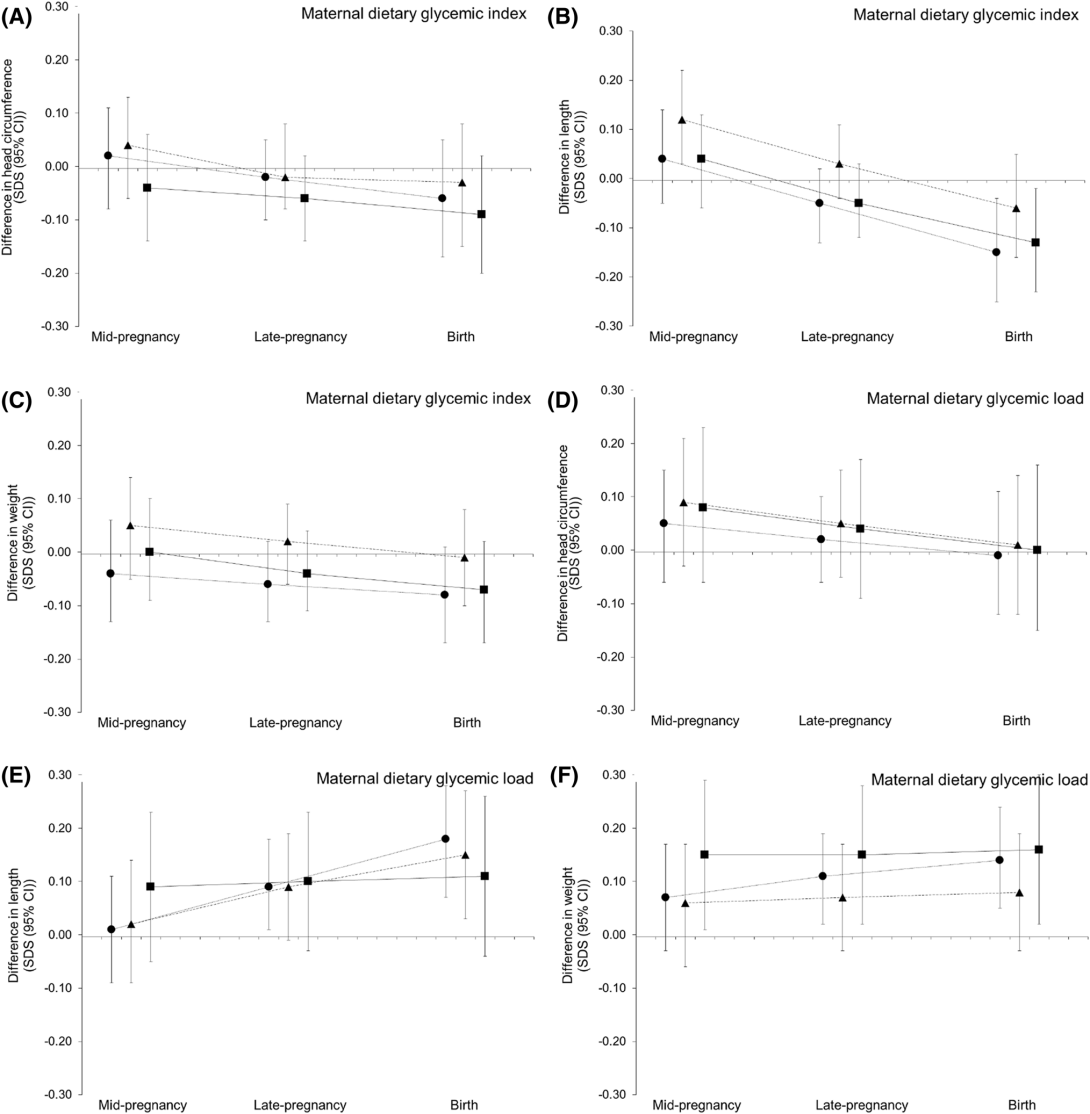
Associations of maternal dietary glycemic index and load with fetal growth patterns from mid-pregnancy onwards differences in fetal growth rates for the upper three maternal dietary glycemic index quartiles (**a**–**c**) and the upper three maternal dietary glycemic load quartiles(**d**–**f**), as compared to the lowest maternal dietary glycemic index and load quartile, respectively. Circles represent the second quartile, triangles the third quartile, and squares the fourth quartile of maternal dietary glycemic index and load, respectively. Results are based on repeated measurement regression models and reflect the differences in gestational-age-adjusted SDS scores of fetal head circumference, length, and weight growth for the three highest maternal dietary glycemic index and load quartiles compared the lowest maternal dietary glycemic index and load quartile (reference group represented as zero line). The models were adjusted for gestational age at study enrollment, maternal age, parity, educational level, pre-pregnancy BMI, early pregnancy total daily energy intake, smoking during pregnancy, alcohol use during pregnancy, daily nausea, and vomiting during early pregnancy and fetal sex. We only observed a significant interaction for maternal dietary glycemic index quartiles with gestational age for fetal length. Regression coefficients for gestational age-independent and gestational age-dependent effects are given in Supplementary Table S3.

Figure 1 shows fetal head circumference, length, and weight growth patterns from mid-pregnancy onwards for quartiles of maternal dietary glycemic index and load. As compared to the lowest quartile of maternal dietary glycemic index, the highest quartile of maternal dietary glycemic index tended to be associated with lower fetal head circumference, length, and weight growth rates from late-pregnancy onwards, but only for fetal length, the *p* value for interaction of maternal dietary glycemic index quartiles with gestational age was significant (*p* value < 0.05). No consistent associations of maternal dietary glycemic load quartiles with fetal growth patterns were present (regression coefficients for gestational age-independent and gestational age-dependent effects in Supplemental Table S3).

Maternal dietary glycemic index within a low-to-normal range was not associated with fetal growth characteristics in each pregnancy period or at birth in the basic or adjusted models (Table 2). In contrast, higher maternal dietary glycemic load within a low-to-normal range was associated with a higher fetal abdominal circumference and fetal estimated weight in late-pregnancy, with stronger associations after adjustment for maternal socio-demographic and lifestyle factors (differences in late-pregnancy fetal abdominal circumference and estimated fetal weight SDS 0.08 [95% CI 0.02, 0.15), 0.07 (95% CI 0.00, 0.14) per SDS increase in glycemic load, respectively]. However, no associations of maternal dietary glycemic load with fetal growth characteristics in mid-pregnancy or at birth were present. Supplemental Table S4 and S5 show that when we analyzed associations of maternal dietary glycemic index and load in quartiles with fetal growth characteristics in each pregnancy period, similar findings were present.

**Table 2:** Associations of maternal early pregnancy dietary glycemic index and load with fetal growth and birth characteristics

	Difference in head circumference SDS (95% CI)	Difference in abdominal circumference SDS (95% CI)	Difference in length SDS (95% CI)	Difference in weight SDS (95% CI)
Maternal early pregnancy glycemic index (SDS)				
	Mid-pregnancy			
	*n* = 3351	*n* = 3354	*n* = 3352	*n* = 3336
Basic model^a^	−0.02 (−0.06; 0.02)	−0.01 (−0.04; 0.02)	0.02 (−0.01; 0.06)	0.01 (−0.03; 0.04)
Adjusted model^b^	−0.01 (−0.05; 0.02)	0.00 (−0.04; 0.03)	0.02 (−0.02; 0.05)	0.01 (−0.03; 0.04)
	Late-pregnancy			
	*n* = 3365	*n* = 3391	*n* = 3400	*n* = 3387
Basic model^a^	−0.03 (−0.06; 0.01)	0.01 (−0.03; 0.04)	−0.02 (−0.05; 0.02)	0.00 (−0.03; 0.04)
Adjusted model^b^	−0.02 (−0.05; 0.02)	0.02 (−0.02; 0.05)	0.00 (−0.04; 0.03)	0.01 (−0.02; 0.05)
	Birth			
	*n* = 1942		*n* = 2323	*n* = 3456
Basic model^a^	−0.03 (−0.08; 0.02)	n.a.	−0.02 (−0.07; 0.02)	−0.03 (−0.06; 0.00)
Adjusted model^b^	−0.02 (−0.07; 0.03)	n.a.	−0.01 (−0.06; 0.04)	−0.02 (−0.06; 0.01)
	Difference in head circumference SDS (95% CI)	Difference in abdominal circumference SDS (95% CI)	Difference in length SDS (95% CI)	Difference in weight SDS (95% CI)
Maternal early pregnancy glycemic load (SDS)				
	Mid-pregnancy			
	*n* = 3351	*n* = 3354	*n* = 3352	*n* = 3336
Basic model^a^	0.01 (−0.02; 0.05)	0.01 (−0.02; 0.05)	0.02 (−0.01; 0.05)	0.02 (−0.01; 0.05)
Adjusted model^b^	0.01 (−0.06; 0.08)	0.03 (−0.04; 0.10)	0.06 (−0.01; 0.12)	0.06 (−0.01; 0.12)
	Late-pregnancy			
	*n* = 3365	*n* = 3391	*n* = 3400	*n* = 3387
Basic model^a^	0.00 (−0.03; 0.04)	0.03 (0.00; 0.07)	0.01 (−0.03; 0.04)	0.03 (0.00; 0.07)
Adjusted model^b^	−0.02 (−0.09; 0.05)	0.08 (0.01; 0.15)*	0.00 (−0.07; 0.07)	0.07 (0.00; 0.14)
	Birth			
	*n* = 1942		*n* = 2323	*n* = 3456
Basic model^a^	0.01 (−0.04; 0.06)	n.a.	0.02 (−0.03; 0.06)	0.02 (−0.02; 0.05)
Adjusted model^b^	0.04 (−0.06; 0.14)	n.a.	0.00 (−0.09; 0.09)	−0.01 (−0.07; 0.06)

n.a.: not available

*p value < 0.05

Values represent regression coefficients (95% confidence interval) from linear regression models that reflect differences in standard deviation score of fetal growth and birth characteristics per one increase in standard deviation of maternal dietary glycemic index and load intake during early pregnancy

^a^Basic models were adjusted for gestational age at study enrollment

^b^Adjusted models were the basic models additionally adjusted for maternal age, parity, educational level, pre-pregnancy BMI, early pregnancy total daily energy intake, smoking during pregnancy, alcohol use during pregnancy, daily nausea, and vomiting during early pregnancy and fetal sex

### Maternal dietary glycemic index and load and the risk of adverse birth outcomes

Higher maternal dietary glycemic index within a low-to-normal range was not associated with the risks of preterm birth, delivering a small-for-gestational-age infant or caesarian delivery (Table 3). Higher maternal dietary glycemic index within a low-to-normal range was associated with a lower risk of delivering a large-for-gestational-age infant in the basic model, which was not explained by adjustment for maternal socio-demographic or lifestyle factors [Odds ratio for the risk of a large-for-gestational-age infant in the adjusted model; 0.86 (95% CI: 0.76, 0.98) per SDS increase in dietary glycemic index]. No associations of maternal dietary glycemic load within a low-to-normal range with adverse birth outcomes were present in the basic or adjusted models. When we analyzed maternal dietary glycemic index and load in quartiles, similar findings were present (Supplemental Table S6 and S7).

**Table 3:** Associations of maternal early pregnancy dietary glycemic index and load with the risks of adverse birth outcomes

	Preterm birth OR (95% CI) (*N *cases = 162)	Small-for-gestational age at birth OR (95% CI) (*N *cases = 345)	Large-for-gestational age at birth OR (95% CI) (*N *cases = 345)	Caesarian delivery OR (95% CI) (*N *cases = 410)
Maternal early pregnancy glycemic index (SDS)				
Basic model^a^	1.13 (0.97; 1.32)	1.04 (0.93; 1.16)	0.86 (0.77; 0.96)*	0.93 (0.84; 1.04)
Adjusted model^b^	1.12 (0.95; 1.32)	1.01 (0.90; 1.14)	0.86 (0.76; 0.97)*	0.98 (0.89; 1.11)
Maternal early pregnancy glycemic load (SDS)				
Basic model^a^	1.01 (0.86; 1.18)	1.03 (0.92; 1.15)	0.94 (0.84; 1.05)	0.93 (0.84; 1.03)
Adjusted model^b^	1.26 (0.92; 1.71)	0.97 (0.78; 1.21)	0.80 (0.64; 1.02)	1.01 (0.82; 1.25)

*p value < 0.05

Values are odds ratios (95% confidence interval) obtained from logistic regression analysis reflecting the differences in odds of adverse birth outcomes per standard deviation change of maternal dietary glycemic index and glycemic load intake during early pregnancy

^a^Basic models were adjusted for gestational age at study enrollment

^b^Adjusted models were the basic models additionally adjusted for maternal age, parity, educational level, pre-pregnancy BMI, early pregnancy total daily energy intake, smoking during pregnancy, alcohol use during pregnancy, daily nausea, and vomiting during early pregnancy and fetal sex

### Sensitivity analyses

In a secondary analysis, no associations of a maternal low-glycemic index diet, based on comparison to individual food product classifications, as compared to a normal-glycemic index diet with fetal growth characteristics or the risks of adverse birth outcomes were present (results not shown). When we excluded women with gestational diabetes, we observed similar results (results not shown). When we repeated the analyses among normal weight women or women aged < 35 years, we observed largely similar effect estimates (Supplemental Tables S8 and S9).

## Discussion

Among pregnant women without an impaired glucose metabolism, we observed that maternal early pregnancy dietary glycemic index across was not associated with fetal growth parameters, whereas a higher maternal early pregnancy dietary glycemic load was associated with a higher fetal abdominal circumference and estimated fetal weight in late-pregnancy only. A higher glycemic index, but not load, was associated with a lower risk of a large-for-gestational-age infant.

There is increasing interest in targeting maternal dietary glycemic index and load during pregnancy as a lifestyle intervention to improve pregnancy and birth outcomes. Small interventions studies among pregnant women with gestational diabetes, impaired glucose tolerance or obesity, have already shown that a lower glycemic index diet from the second half of pregnancy onwards improves maternal glucose concentrations and lowers the risk of delivering a large-for-gestational-age infant [19, 34]. With dietary interventions, these studies achieved a median maternal dietary glycemic index around 50 or lower in their intervention groups and compared these effects to a normal or high maternal dietary glycemic index. Far less is known about the effects of maternal dietary glycemic index and load on birth outcomes among populations not at risk for an impaired glucose metabolism.

A few previous studies focused on the associations of maternal dietary glycemic index and load with birth characteristics and the risks of adverse birth outcomes among general, healthy populations, but no studies focused on directly measured fetal growth characteristics [9, 17, 18, 30]. These studies differed strongly with regards to the methods used to calculate maternal dietary glycemic index and load, the timing of the dietary assessments, studied populations, and adjustment for maternal socio-demographic and lifestyle characteristics. An observational study among 47,003 Danish pregnant women reported that a higher maternal dietary glycemic load, but not index, in mid-pregnancy was associated with a higher birth weight and an increased risk of delivering a large-for-gestational-age infant [30]. A study among 1,082 multi-ethnic non-diabetic pregnant women from USA showed that the lowest quintile of maternal mid-pregnancy dietary glycemic index, but not load, was associated with a lower birth weight and an increased risk of delivering a small-for-gestational-age infant. Using white bread instead of glucose as a reference, the glycemic index in this study varied < 71 for the lowest quintile to > 85 for the highest quintile. No associations of the highest quintile of maternal mid-pregnancy dietary glycemic index or load with a higher birth weight and increased risk of delivering a large-for gestational-age-infant were observed [9]. Contrarily, a study among 842 low-risk Irish pregnant women reported no associations of maternal dietary glycemic index and load in early pregnancy continuously with birth weight or adverse birth outcomes, after adjusting for maternal age, pre-pregnancy BMI, and parity and considering multiple testing [17]. Similarly, a study among 906 low-risk pregnant women from the UK showed no associations of maternal early or late-pregnancy dietary glycemic index and load continuously with fat and lean mass at birth [18]. The mean and variability of the glycemic index in these two studies were comparable to ours.

In line with these previous studies focused on maternal early pregnancy dietary glycemic index and load, we observed that women within our study consumed diet with a relatively low mean dietary glycemic index. No consistent associations of maternal early pregnancy dietary glycemic index and load across the low-to-normal range with birth weight and the risks of adverse birth outcomes were observed. We did observe that a higher maternal early pregnancy dietary glycemic load, especially within the highest quartile, was associated with a higher late-pregnancy fetal abdominal circumference and estimated fetal weight, but findings were not consistent across pregnancy and may reflect a chance finding. However, fetal fat development mainly occurs in late-pregnancy and abdominal circumference is an important indicator of fetal fat deposition [35]. This could suggest that a higher maternal early pregnancy dietary glycemic load may rather affect fetal body composition than growth, which is also suggested by the previous studies conducted in infants [36, 37].

Contrary to our prior hypothesis, we observed that a higher maternal early pregnancy dietary glycemic index within a low-to-normal range was associated with lower fetal length growth rates from late-pregnancy onwards and with a lower risk of delivering a large-for-gestational-age infant only. These association were not explained by maternal socio-demographic and lifestyle characteristics. It could reflect a chance finding. Our study population is a relatively healthy population not at high risk of an impaired glucose tolerance. We only included Dutch women without pre-gestational diabetes and we observed largely similar results for women with a normal weight, younger than 35 years old, and without gestational diabetes. Possibly, the range of maternal dietary glycemic index within our population reflects a relatively healthy range for women at a low risk of an impaired glucose metabolism in early pregnancy. Maternal dietary glycemic index within this range may be not related to increased risks of fetal undergrowth or overgrowth. The timing of dietary glycemic index assessment in early pregnancy may also be important. Maternal insulin sensitivity is much higher in early pregnancy as compared to mid- and late-pregnancy, which leads to smaller fluctuations in postprandial glycemic responses to carbohydrate containing foods in early pregnancy [5]. Potential adverse effects of a higher maternal dietary glycemic index on fetal growth and the risk of macro-somia may be more pronounced in the second half of pregnancy, when pregnant women are physiologically more insulin resistant and the postprandial glycemic response shows larger fluctuations. Finally, postprandial peaks in maternal glucose concentrations and subsequent peak increases in fetal glucose concentrations may rather have an effect on fetal body composition and fetal metabolism than on skeletal growth, by affecting fetal development of adipocytes and the cardio-metabolic system [18, 36, 37]. This hypothesis is supported by the associations which we observed of a higher maternal early pregnancy glycemic load with fetal abdominal circumference and estimated fetal weight in late-pregnancy when fetal fat accumulation occurs. Further studies using multiple assessments of dietary intake throughout pregnancy are needed to examine the detailed associations of maternal dietary glycemic index and load with both fetal and neonatal growth and body composition.

Importantly, in a secondary analysis, we observed no increased risks of preterm birth, small-for-gestational-age at birth, or caesarian delivery, as complication of abnormal fetal growth, among women consuming a low-glycemic index diet, as compared to women consuming a normal-glycemic index diet. The mean dietary glycemic index of women consuming a low-glycemic index diet within our study was largely similar to the mean dietary glycemic index reported in intervention studies stimulating a low-glycemic index diet through advising low-glycemic index food products [19, 21]. This suggests that even among pregnant populations without an impaired glucose metabolism, a diet with lower glycemic index products in early pregnancy does not appear to be associated with fetal growth restriction and related adverse birth outcomes. These findings are important from a public health perspective, as there is an increasing interest in stimulating a diet with low-glycemic index products during pregnancy to improve birth and childhood outcomes. Our findings suggest that adhering to a diet with low-glycemic index products may be a safe intervention during pregnancy without adverse effects on fetal growth and birth outcomes in women without an impaired glucose metabolism. The beneficial effects of a lower dietary glycemic index and load within general, healthy populations on fetal growth and birth outcomes remain to be determined.

### Strengths and limitations

Strengths of this study were the prospective study design, large sample size, and repeatedly measured fetal growth data from mid-pregnancy onwards available. Limitations of this study should also be taken into account when interpreting results. First, the response rate at baseline for participating in the Generation R study cohort was 61%. The non-response would have led to biased effect estimates if the associations were different between those included and not included in the analyses. However, this seems unlikely because biased estimates in large cohort studies often arise from loss to follow-up rather than from non-response at baseline [38]. Second, we did not have information on previous gestational diabetes or polycystic ovarian syndrome, which are also associated with an increased risk of an impaired glucose metabolism. Although we expect the number of cases of previous gestational diabetes and polycystic ovarian syndrome to be low, as we had a relatively healthy population, this may have affected our results. Further studies excluding these women should replicate our findings. The selection towards a relatively healthy Dutch population may affect the generalizability of our findings and might have led to reduced statistical power. Most women had a dietary glycemic index and load within the normal range and the number of adverse birth outcomes was also relatively low. Further studies are needed among multi-ethnic populations with a more diverse dietary intake to replicate our findings. Third, even though the FFQ is widely used for dietary assessment in observational studies, measurement of food intake by an FFQ may be affected by measurement error, recall bias, and reporting bias. Subsequent calculation of the dietary glycemic index and load from the FFQ may further be affected by uncertainty induced by preparation of foods, mixed dishes, variations of food products of time, or unavailability of specific food products [20]. Fourth, we obtained information on maternal dietary intake only once during pregnancy. Further studies from preconception onwards are needed using repeated assessments of maternal dietary intake prior and throughout pregnancy to obtain further insight into critical periods for the influence of maternal dietary glycemic quality and quantity on embryonic and fetal development and adverse birth outcomes. Finally, although were able to adjust for multiple confounding factors, there might still be residual confounding as in any observational study.

## Conclusion

Among pregnant women without an impaired glucose metabolism, a higher maternal early pregnancy dietary glycemic load was associated with a higher fetal abdominal circumference and estimated fetal weight in late-pregnancy. Maternal dietary glycemic index and load were not consistently associated with fetal growth parameters in mid- pregnancy and at birth. A higher glycemic index was associated with a lower risk of a large-for-gestational-age infant. Further studies with a larger variability in maternal dietary glycemic index and load among multi-ethnic low-risk populations are needed to assess whether a lower glycemic index diet is a feasible lifestyle intervention to improve fetal growth and birth outcomes.

## Electronic supplementary material

Below is the link to the electronic supplementary material.

Electronic supplementary material 1 (DOCX 57 kb)
