# Supplementary material for: Maternal early pregnancy dietary glycemic index and load, fetal growth, and the risk of adverse birth outcomes
Source: Eur J Nutr. 2020 Jul 14;60(3):1301–11. doi: 10.1007/s00394-020-02327-9 (PMC7987612; doi:10.1007/s00394-020-02327-9)
Supplement: Supplementary file 1 — Electronic supplementary material 1 (DOCX 57 kb) [file 394_2020_2327_MOESM1_ESM.docx]

**Supplemental Material**

Maternal early-pregnancy dietary glycemic index, fetal growth and the risk of adverse birth outcomes

*Running title: Maternal dietary glycemic index and fetal growth*

Rama J. Wahab MD^1,2^, Judith M. Scholing^1,2,3^, Romy Gaillard MD PhD^1,2^.

1. The Generation R Study Group, Erasmus MC, University Medical Center, Rotterdam, the Netherlands.
2. Department of Pediatrics, Sophia’s Children’s Hospital, Erasmus MC, University Medical Center, Rotterdam, the Netherlands.
3. Division of Human Nutrition and Health, Wageningen University & Research, Wageningen, the Netherlands.

**Content**

**Figure S1** Flow chart of the study population

**Table S1** Fetal growth characteristics according to maternal dietary glycemic index quartiles

**Table S2** Non-response analysis for women with a

singleton live birth and information dietary intake during early-pregnancy, compared to women with a singleton live birth without data on dietary intake during early-pregnancy

**Table S3** Regression coefficients of longitudinal associations between maternal dietary glycemic index quartiles with fetal growth patterns

**Table S4** Associations of maternal dietary glycemic index in quartiles with fetal and birth characteristics

**Table S5** Associations of maternal dietary glycemic load in quartiles with fetal and birth characteristics

**Table S6** Associations of maternal dietary glycemic index in quartiles with the risk of adverse birth outcomes

**Table S7** Associations of maternal dietary glycemic load in quartiles with the risk of adverse birth outcomes

**Table S8** Associations of maternal dietary glycemic index and load in women with a BMI <25 kg/m^2^ with fetal and birth parameters

**Table S9** Associations of maternal dietary glycemic index and load in women aged <35 years with fetal and birth parameters

**Supplemental Figure S1. Flow chart of the study population**

**n= 4,544**

Pregnant women of Dutch ethnicity enrolled during pregnancy

**n= 3,558**

Mothers with information available on dietary intake

**n=3,471**

**Population for analysis:**

Dutch mothers with singleton live births with information available on dietary intake and fetal growth and/or birth outcomes.

Fetal growth characteristics

Mid-pregnancy **n=3,373**

Late-pregnancy **n=3,404**

Birth characteristics **n=3,471**

**n= 87**

**Excluded:**

Multiple pregnancy n= 53

Induced abortion n= 8

Intrauterine fetal death n= 16

Loss to follow up n= 3

Pregestational diabetes n=7

**n= 986**

**Excluded:**

No information or non-reliable information available on dietary intake in early-pregnancy

**Supplemental Table S1. Fetal growth characteristics according to maternal dietary glycemic index quartiles**

|  | Total group | Glycemic index quartile 1 | Glycemic index quartile 2 | Glycemic index quartile 3 | Glycemic index quartile 4 | P-value^a^ |
| --- | --- | --- | --- | --- | --- | --- |
| Mid-pregnancy |  |  |  |  |  |  |
| Gestational age at ultrasound measurement, mean (SD) | 20.6 (1.1) | 20.6 (1.1) | 20.6 (1.1) | 20.6 (1.0) | 20.7 (1.1) | 0.13 |
| Head circumference, mean (SD), mm | 179 (14) | 180 (14) | 179 (14) | 179 (13) | 180 (13) | 0.56 |
| Abdominal circumference, mean (SD), mm | 157 (14) | 157 (15) | 157 (13) | 156 (14) | 158 (14) | 0.22 |
| Femur length, mean (SD), mm | 33 (3) | 33 (3) | 33 (3) | 33 (3)_ | 34 (3) | 0.43 |
| Estimated fetal weight, mean (SD), g | 379 (87) | 381 (93) | 376 (84) | 378 (84) | 383 (86) | 0.35 |
| Late-pregnancy |  |  |  |  |  |  |
| Gestational age at ultrasound measurement, mean (SD) | 30.4 (1.0) | 30.5 (1.0) | 30.5 (1.0) | 30.4 (1.0) | 30.4 (1.1) | 0.12 |
| Head circumference, mean (SD), mm | 286 (12) | 287 (12) | 286 (12) | 285 (13) | 285 (12) | 0.10 |
| Abdominal circumference, mean (SD), mm | 265 (16) | 266 (16) | 265 (16) | 265 (17) | 265 (17) | 0.29 |
| Femur length, mean (SD), mm | 57 (3) | 58 (3) | 57 (3) | 57 (3) | 57 (3) | 0.27 |
| Estimated fetal weight, mean (SD), g | 1633 (259) | 1646 (249) | 1627 (258) | 1630 (269) | 1628 (259) | 0.39 |

^a^P-values were obtained by ANOVA-tests

**Supplemental Table S2. Non-response analysis for women with a singleton live birth and information dietary intake during early-pregnancy, compared to women with a singleton live birth without data on dietary intake during early-pregnancy**

|  | | Women with information on dietary glycemic index (n=3,471) | Women without information on dietary glycemic index (n=924) | P-value^a^ |  |
| --- | --- | --- | --- | --- | --- |
| Maternal characteristics | |  |  |  |  |
| Maternal age at enrolment, mean (SD), years | | 31.4 (4.4) | 31.7 (5.2) | 0.09 | |
| Parity, n nulliparous (%) | | 2,076 (59.9) | 379 (46.1) | <0.01 | |
| Pre-pregnancy BMI group, n (%) | |  |  | 0.08 | |
|  | Underweight | 25 (5.7) | 25 (5.8) |  | |
|  | Normal weight | 306 (70.2) | 305 (71.1) |  | |
|  | Overweight | 72 (16.5) | 70 (16.3) |  | |
|  | Obese | 33 (7.6) | 29 (6.8) |  | |
| Gestational weight gain, mean (SD), g/week | | 10.8 (4.4) | 11.2 (4.8) | 0.08 | |
| Education, n high (%) | | 2,026 (59.1) | 464 (52.5) | <0.01 | |
| Folic acid supplement use, n yes (%) | | 2,532 (72.9) | 342 (82.0) | <0.01 | |
| Alcohol use during pregnancy, yes (%) | | 2,117 (66.3) | 275 (58.8) | <0.01 | |
| Smoking during pregnancy, n (%) | | 833 (26.9) | 213 (26.1) | 0.19 | |
| Nausea during early-pregnancy, n (%) | | 880 (27.7) | 141 (30.7) | 0.23 | |
| Vomiting during early-pregnancy, n (%) | | 145 (4.6) | 37 (8.1) | <0.01 | |
| Gestational diabetes, n (%) | | 31 (0.9) | 13 (1.5) | 0.12 | |
| Child characteristics | |  |  |  | |
| Sex, n (%) male | | 1,753 (50.5) | 493 (51.5) | 0.58 | |
| Gestational age at birth, median (95%) | | 40.3 (37.0, 42.1) | 40.0 (36.7, 42.0) | <0.01 | |
| Birthweight, mean (SD), g | | 3,489 (554) | 3445 (593) | 0.04 | |

BMI: body mass index.

^a^P-values were obtained by independent t-test or Mann-Whitney U-test for continuous variables and chi-square tests for categorical variables.

**Supplemental Table S3. Regression coefficients of longitudinal associations between maternal dietary glycemic index quartiles with fetal growth patterns^a^**

|  | Intercept  Head circumference  (SDS) | Slope  Head circumference (SDS) | Intercept  Length  (SDS) | Slope  Length (SDS) | Intercept  Weight  (SDS) | Slope  Weight (SDS) |
| --- | --- | --- | --- | --- | --- | --- |
| Maternal dietary glycemic index |  |  |  |  |  |  |
| Quartile 1 | Ref. | Ref. | Ref. | Ref. | Ref. | Ref. |
| Quartile 2 | 0.096 (-0.153; 0.281) | -0.004 (-0.011; 0.003) | 0.229 (0.029; 0.429) | -0.009 (-0.012; -0.005) | 0.007 (-0.167; 0.180) | -0.002 (-0.007; 0.003) |
| Quartile 3 | 0.108 (-0.111; 0.0.326) | -0.004 (-0.011; 0.004) | 0.300 (0.100; 0.500) | -0.009 (-0.015; -0.025) | 0.103 (-0.071; 0.278) | -0.003 (-0.008; 0.002) |
| Quartile 4 | 0.018 (-0.200; 0.235) | -0.003 (-0.010; 0.004) | 0.200 (-0.001; 0.400) | -0.008 (-0.015; -0.002) | 0.076 (-0.100; 0.251) | -0.004 (-0.009; 0.001) |
| Maternal dietary glycemic load |  |  |  |  |  |  |
| Quartile 1 | Ref. | Ref. | Ref. | Ref. | Ref. | Ref. |
| Quartile 2 | 0.103 (-0.117; 0.323) | -0.003 (-0.010; 0.004) | -0.149 (-0.352; 0.054) | 0.008 (0.001; 0.012) | 0.004 (-0.173; 0.182) | 0.003 (-0.002; 0.009) |
| Quartile 3 | 0.168 (-0.059; 0.394) | -0.004 (-0.011; 0.003) | -0.104 (-0.314; 0.107) | 0.006 (0.000; 0.013) | 0.032 (-0.153; 0.218) | 0.001 (-0.004; 0.006) |
| Quartile 4 | 0.162 (-0.080; 0.405) | -0.004 (-0.011; 0.003) | 0.078 (-0.149; 0.304) | 0.001 (-0.006; 0.007) | 0.137 (-0.067; 0.342) | 0.001 (-0.005; 0.006) |

^a^Values are regression coefficients obtained from linear repeated measurement models and reflect the (gestational)

age independent differences (intercepts) and the gestational age dependent differences (slopes: change in growth characteristics SDS per week per quartile of dietary glycemic index and glycemic load intake during early-pregnancy, compared with the lowest quartile of maternal dietary glycemic index and load as the reference group adjusted for gestational age at study enrolment, maternal age, parity, pre-pregnancy BMI, maternal education, smoking during pregnancy, alcohol use during pregnancy, nausea during early-pregnancy, vomiting during early-pregnancy, early-pregnancy total daily energy intake and fetal sex

**Supplemental Table S4. Associations of maternal dietary glycemic index in quartiles with fetal and birth characteristics^a^**

| Maternal dietary glycemic index | Head circumference (SDS) | | Abdominal circumference (SDS) | | Length (SDS) | | Weight (SDS) | |
| --- | --- | --- | --- | --- | --- | --- | --- | --- |
|  | Basic model^b^ | Adjusted model^c^ | Basic model^b^ | Adjusted model^c^ | Basic model^b^ | Adjusted model^c^ | Basic model^b^ | Adjusted model^c^ |
|  | ***Mid pregnancy*** | | | | | | | |
| Quartile 1 | Ref. | Ref. | Ref. | Ref. | Ref. | Ref. | Ref. | Ref. |
| Quartile 2 | 0.04 (-0.06; 0.14) | 0.03 (-0.07; 0.12) | -0.06 (-0.16; 0.04) | -0.06 (-0.16; 0.04) | 0.02 (-0.07; 0.12) | 0.02 (-0.08; 0.11) | -0.04 (-0.14; 0.06) | -0.04 (-0.14; 0.05) |
| Quartile 3 | 0.03 (-0.07; 0.13) | 0.05 (-0.05; 0.15) | -0.04 (-0.14; 0.06) | -0.03 (-0.12; 0.07) | 0.15 (0.05; 0.24) | 0.12 (0.02; 0.22)* | 0.05 (-0.05; 0.15) | 0.05 (-0.05; 0.14) |
| Quartile 4 | -0.05 (-0.15; 0.05) | -0.04 (-0.14; 0.06) | -0.05 (-0.15; 0.04) | -0.03 (-0.13; 0.07) | 0.03 (-0.06; 0.13) | 0.02 (-0.08; 0.11) | -0.02 (-0.11; 0.08) | -0.01 (-0.11; 0.09) |
|  | ***Late pregnancy*** | | | | | | | |
| Quartile 1 | Ref. | Ref. | Ref. | Ref. | Ref. | Ref. | Ref. | Ref. |
| Quartile 2 | -0.04 (-0.14; 0.06) | -0.06 (-0.15; 0.04) | -0.09 (-0.19; 0.01) | -0.09 (-0.19; 0.01) | -0.03 (-0.13; 0.06) | -0.02 (-0.12; 0.07) | -0.08 (-0.18; 0.02) | -0.08 (-0.17; 0.02) |
| Quartile 3 | -0.03 (-0.12; 0.07) | 0.00 (-0.10; 0.09) | -.0.01 (-0.11; 0.09) | 0.01 (-0.10; 0.09) | 0.03 (-0.07; 0.12) | 0.03 (-0.07; 0.12) | 0.01 (-0.09; 0.11) | 0.01 (-0.08; 0.11) |
| Quartile 4 | -0.09 (-0.19; 0.00) | -0.07 (-0.17; 0.03) | -0.04 (-0.13; 0.06) | -0.02 (-0.11; 0.08) | -0.07 (-0.17; 0.02) | -0.05 (-0.15; 0.04) | -0.06 (-0.15; 0.04) | -0.03 (-0.13; 0.07) |
|  | ***Birth*** | | | | | | | |
| Quartile 1 | Ref. | Ref. | n.a. | n.a. | Ref. | Ref. | Ref. | Ref. |
| Quartile 2 | -0.03 (-0.17; 0.12) | -0.02 (-0.16; 0.12) | n.a. | n.a. | -0.19 (-0.32; -0.06)* | -0.19 (-0.32; -0.06)* | -0.07 (-0.16; 0.03) | -0.07 (-0.16; 0.03) |
| Quartile 3 | -0.07 (-0.21; 0.07) | -0.07 (-0.21; 0.08) | n.a. | n.a. | -0.08 (-0.21; 0.05) | -0.07 (-0.20; 0.06) | 0.00 (-0.10; 0.10) | -0.01 (-0.11; 0.08) |
| Quartile 4 | -0.09 (-0.23; 0.05) | -0.07 (-0.22; 0.07) | n.a. | n.a. | -0.13 (-0.26; 0.00)* | -0.09 (-0.23; 0.04) | -0.09 (-0.18; 0.01) | -0.07 (-0.16; 0.03) |

*P<0.05.

^a^Values represent regression coefficients (95% confidence interval) from linear regression models that reflect differences in standard deviation change of fetal and birth growth parameters per quartile of maternal dietary glycemic index during early-pregnancy as compared to the lowest quartile

^b^Basic models were adjusted for gestational age at study enrolment

^c^Adjusted models were the basic models additionally adjusted for maternal age, parity, educational level, prepregnancy BMI, early-pregnancy total daily energy intake, smoking during pregnancy, alcohol use during pregnancy, daily nausea and vomiting during early-pregnancy and fetal sex

**Supplemental Table S5. Associations of maternal dietary glycemic load in quartiles with fetal and birth characteristics^a^**

| Maternal dietary glycemic load | Head circumference (SDS) | | Abdominal circumference (SDS) | | Length (SDS) | | Weight (SDS) | |
| --- | --- | --- | --- | --- | --- | --- | --- | --- |
|  | Basic model^b^ | Adjusted model^c^ | Basic model^b^ | Adjusted model^c^ | Basic model^b^ | Adjusted model^c^ | Basic model^b^ | Adjusted model^c^ |
|  | **Mid pregnancy** | | | | | | | |
| Quartile 1 | Ref. | Ref. | Ref. | Ref. | Ref. | Ref. | Ref. | Ref. |
| Quartile 2 | 0.06 (-0.04; 0.15) | 0.07 (-0.05; 0.18) | 0.13 (0.03; 0.22)* | 0.15 (0.04; 0.26)* | 0.03 (-0.06; 0.13) | 0.06 (-0.05; 0.16) | 0.10 (0.01; 0.20)* | 0.13 (0.03; 0.24)* |
| Quartile 3 | 0.09 (-0.01; 0.19) | 0.09 (-0.01; 0.25) | 0.06 (-0.04; 0.15) | 0.10 (-0.02; 0.23) | 0.02 (-0.08; 0.11) | 0.07 (-0.06; 0.16) | 0.04 (-0.05; 0.14) | 0.11 (-0.02; 0.23) |
| Quartile 4 | 0.06 (-0.04; 0.16) | 0.10 (-0.07; 0.30) | 0.09 (-0.01; 0.19) | 0.19 (0.02; 0.35)* | 0.06 (-0.03; 0.16) | 0.14 (-0.03; 0.30) | 0.10 (0.00; 0.19)* | 0.21 (0.04; 0.37)* |
|  | **Late pregnancy** | | | | | | | |
| Quartile 1 | Ref. | Ref. | Ref. | Ref. | Ref. | Ref. | Ref. | Ref. |
| Quartile 2 | 0.07 (-0.03; 0.17) | 0.04 (-0.07; 0.14) | 0.07 (-0.02; 0.17) | 0.07 (-0.04; 0.18) | 0.09 (-0.01; 0.18) | 0.08 (-0.03; 0.18) | 0.09 (-0.01; 0.19) | 0.09 (-0.02; 0.20) |
| Quartile 3 | 0.07 (-0.03; 0.17) | 0.03 (-0.09; 0.16) | 0.03 (-0.07; 0.13) | 0.05 (-0.07; 0.18) | 0.05 (-0.04; 0.14) | 0.06 (-0.06; 0.18) | 0.04 (-0.05; 0.14) | 0.07 (-0.06; 0.20) |
| Quartile 4 | 0.01 (-0.08; 0.11) | -0.01 (-0.17; 0.15) | 0.10 (0.00; 0.19) | 0.16 (0.00; 0.33)* | 0.05 (-0.05; 0.14) | 0.07 (-0.09; 0.23) | 0.10 (0.00; 0.20) | 0.17 (0.00; 0.34)* |
|  | **Birth** | | | | | | | |
| Quartile 1 | Ref. | Ref. | n.a. | n.a. | Ref. | Ref. | Ref. | Ref. |
| Quartile 2 | -0.02 (-0.16; 0.12) | -0.05 (-0.20; 0.11) | n.a. | n.a. | 0.19 (0.06; 0.32)* | 0.14 (-0.01; 0.28) | 0.16 (0.06; 0.25)* | 0.11 (0.00; 0.21) |
| Quartile 3 | 0.00 (-0.15; 0.14) | -0.02 (-0.21; 0.17) | n.a. | n.a. | 0.17 (0.03; 0.30)* | 0.13 (-0.05 ;0.30) | 0.06 (-0.04; 0.15) | 0.02 (-0.10; 0.14) |
| Quartile 4 | -0.02 (-0.16; 0.12) | -0.03 (-0.27; 0.22) | n.a. | n.a. | 0.08 (-0.05; 0.21) | 0.06 (-0.17; 0.28) | 0.10 (0.00; 0.20)* | 0.07 (-0.09; 0.23) |

*P<0.05.

^a^Values represent regression coefficients (95% confidence interval) from linear regression models that reflect differences in standard deviation change of fetal and birth growth parameters per quartile of maternal dietary glycemic load during early-pregnancy as compared to the lowest quartile

^b^Basic models were adjusted for gestational age at study enrolment

^c^Adjusted models were the basic models additionally adjusted for maternal age, parity, educational level, prepregnancy BMI, early-pregnancy total daily energy intake, smoking during pregnancy, alcohol use during pregnancy, daily nausea and vomiting during early-pregnancy and fetal sex

**Supplemental Table S6. Associations of maternal dietary glycemic index in quartiles with the risk of adverse birth outcomes^a^**

| Maternal dietary glycemic index | Preterm birth  OR (95% CI)  *(Ncases=162)* | Small-for-gestational age at birth  OR (95% CI)  *(Ncases =345)* | Large-for-gestational  age at birth  OR (95% CI)  *(Ncases =345)* | Caesarian section  OR (95% CI)  *(Ncases =410)* |
| --- | --- | --- | --- | --- |
| Basic model^b^ |  |  |  |  |
| Quartile 1 | Ref. | Ref. | Ref. | Ref |
| Quartile 2 | 0.78 (0.49; 1.23) | 0.96 (0.70; 1.33) | 0.79 (0.58; 1.07) | 0.83 (0.62; 1.11) |
| Quartile 3 | 0.68 (0.43; 1.10) | 1.03 (0.75; 1.41) | 0.83 (0.62; 1.12) | 0.78 (0.58; 1.04) |
| Quartile 4 | 1.30 (0.87; 1.97) | 1.07 (0.79; 1.47) | 0.60 (0.43; 0.83)* | 0.84 (0.63; 1.12) |
| Adjusted model^c^ |  |  |  |  |
| Quartile 1 | Ref. | Ref. | Ref. | Ref. |
| Quartile 2 | 0.76 (0.48; 1.21) | 0.95 (0.69; 1.32) | 0.77 (0.57; 1.06) | 0.86 (0.64; 1.15) |
| Quartile 3 | 0.68 (0.42; 1.11) | 1.03 (0.74; 1.42) | 0.81 (0.60; 1.11) | 0.85 (0.63; 1.15) |
| Quartile 4 | 1.28 (0.83; 1.98) | 1.01 (0.73; 1.41) | 0.60 (0.43; 0.84)* | 1.00 (0.74; 1.135) |

*P<0.05.

^a^Values are odds ratios (95% Confidence Interval) obtained from logistic regression analysis reflecting the differences in odds of adverse birth outcomes per quartile of maternal dietary glycemic index during early-pregnancy as compared to the lowest quartile

^b^Basic models were adjusted for gestational age at study enrolment

^c^Adjusted models were the basic models additionally adjusted for maternal age, parity, educational level, prepregnancy BMI, early-pregnancy total daily energy intake, smoking during pregnancy, alcohol use during pregnancy, daily nausea and vomiting during early-pregnancy and fetal sex

**Supplemental Table S7. Associations of maternal dietary glycemic load in quartiles with the risk of adverse birth outcomes^a^**

| Maternal dietary glycemic load | Preterm birth  OR (95% CI)  *(Ncases=162)* | Small-for-gestational age at birth  OR (95% CI)  *(Ncases =345)* | Large-for-gestational  age at birth  OR (95% CI)  *(Ncases =345)* | Caesarian section  OR (95% CI)  *(Ncases =410)* |
| --- | --- | --- | --- | --- |
| Basic model^b^ |  |  |  |  |
| Quartile 1 | Ref. | Ref. | Ref. | Ref. |
| Quartile 2 | 0.71 (0.45; 1.13) | 0.79 (0.58; 1.09) | 1.10 (0.81; 1.50) | 0.85 (0.64; 1.13) |
| Quartile 3 | 0.98 (0.63; 1.50) | 0.89 (0.65; 1.22) | 0.88 (0.63; 1.21) | 0.74 (0.55; 0.99) |
| Quartile 4 | 0.96 (0.62; 1.48) | 1.01 (0.75; 1.38) | 0.99 (0.72; 1.35) | 0.80 (0.60; 1.07) |
| Adjusted model^c^ |  |  |  |  |
| Quartile 1 | Ref. | Ref. | Ref. | Ref. |
| Quartile 2 | 0.88 (0.52; 1.48) | 0.80 (0.56; 1.16) | 1.00 (0.70; 1.42) | 0.87 (0.62; 1.20) |
| Quartile 3 | 1.34 (0.75; 2.39) | 0.82 (0.54; 1.25) | 0.80 (0.52; 1.23) | 0.76 (0.51; 1.12) |
| Quartile 4 | 1.55 (0.73; 3.33) | 0.85 (0.49; 1.46) | 0.90 (0.52; 1.57) | 0.90 (0.53; 1.50) |

*P<0.05.

^a^Values are odds ratios (95% Confidence Interval) obtained from logistic regression analysis reflecting the differences in odds of adverse birth outcomes per quartile of maternal dietary glycemic load during early-pregnancy as compared to the lowest quartile

^b^Basic models were adjusted for gestational age at study enrolment

^c^Adjusted models were the basic models additionally adjusted for maternal age, parity, educational level, prepregnancy BMI, early-pregnancy total daily energy intake, smoking during pregnancy, alcohol use during pregnancy, daily nausea and vomiting during early-pregnancy and fetal sex

**Table S8. Associations of maternal dietary glycemic index and load in women with a BMI <25 kg/m^2^ with fetal and birth parameters**

|  | Difference in head circumference SDS (95% CI) | Difference in abdominal circumference SDS (95% CI) | Difference in length  SDS (95% CI) | Difference in weight  SDS (95% CI) |
| --- | --- | --- | --- | --- |
| Maternal early-pregnancy glycemic index (SDS) |  |  |  |  |
|  | **Mid-pregnancy** | | | |
|  | *n=2533* | *n=2538* | *n=2539* | *n=2528* |
| Basic model^b^ | -0.01 (-0.05, 0.03) | 0.00 (-0.04, 0.04) | 0.02 (-0.01, 0.06) | 0.02 (-0.02, 0.06) |
| Adjusted model^c^ | -0.01 (-0.05, 0.04) | 0.01 (-0.03, 0.05) | 0.02 (-0.02; 0.05) | 0.02 (-0.02, 0.06) |
|  | **Late-pregnancy** | | | |
|  | *n=2535* | *n=2559* | *n=2563* | *n=2555* |
| Basic model^b^ | -0.02 (-0.06, 0.02) | 0.00 (-0.04, 0.04) | -0.01 (-0.05, 0.03) | 0.00 (-0.04, 0.04) |
| Adjusted model^c^ | -0.01 (-0.05, 0.03) | 0.02 (-0.02, 0.06) | 0.01 (-0.03, 0.05) | 0.02 (-0.02, 0.07) |
|  | **Birth** | | | |
|  | *n=1479* |  | *n=1757* | *n=2605* |
| Basic model^b^ | -0.01 (-0.07, 0.05) | n.a. | 0.00 (-0.06, 0.05) | -0.01 (-0.06; 0.03) |
| Adjusted model^c^ | -0.00 (-0.06, 0.07) | n.a. | 0.02 (-0.04, 0.07) | -0.01 (-0.05, 0.03) |
|  | **Difference in head circumference SDS (95% CI)** | **Difference in abdominal circumference SDS (95% CI)** | **Difference in length**  **SDS (95% CI)** | **Difference in weight**  **SDS (95% CI)** |
| Maternal early-pregnancy glycemic load (SDS) |  | | | |
|  | **Mid-pregnancy** | | | |
|  | *n=2533* | *n=2538* | *n=2539* | *n=2528* |
| Basic model^b^ | 0.03 (-0.01, 0.08) | 0.03 (-0.01, 0.07) | 0.03 (-0.01, 0.07) | 0.04 (-0.01, 0.08) |
| Adjusted model^c^ | 0.04 (-0.04, 0.12) | 0.06 (-0.02, 0.13) | 0.09 (0.02, 0.17)* | 0.09 (0.02, 0.17)* |
|  | **Late-pregnancy** | | | |
|  | *n=2535* | *n=2559* | *n=2563* | *n=2555* |
| Basic model^b^ | 0.02 (-0.03, 0.06) | 0.05 (0.01, 0.08)* | 0.01 (-0.03, 0.05) | 0.04 (0.00, 0.08)* |
| Adjusted model^c^ | 0.01 (-0.07, 0.09) | 0.11 (0.03, 0.19)* | 0.03 (-0.05, 0.10) | 0.10 (0.02, 0.18)* |
|  | **Birth** | | | |
|  | *n=1479* |  | *n=1757* | *n=2605* |
| Basic model^b^ | 0.03 (-0.03; 0.09) | n.a. | 0.04 (-0.02, 0.09) | 0.02 (-0.02, 0.06) |
| Adjusted model^c^ | 0.09 (-0.03, 0.21) | n.a. | 0.03 (-0.08, 0.14) | 0.03 (-0.05, 0.11) |

n.a.: not available

*P<0.05

^a^Values represent regression coefficients (95% confidence interval) from linear regression models that reflect differences in standard deviation score of fetal growth and birth characteristics per one increase in standard deviation of maternal dietary glycemic index and load intake during early-pregnancy

^b^Basic models were adjusted for gestational age at study enrolment

^c^Adjusted models were the basic models additionally adjusted for maternal age, parity, educational level, prepregnancy BMI, early-pregnancy total daily energy intake, smoking during pregnancy, alcohol use during pregnancy, daily nausea and vomiting during early-pregnancy and fetal sex

**Table S9. Associations of maternal dietary glycemic index and load in women aged <35 years with fetal and birth parameters**

|  | Difference in head circumference SDS (95% CI) | Difference in abdominal circumference SDS (95% CI) | Difference in length  SDS (95% CI) | Difference in weight  SDS (95% CI) |
| --- | --- | --- | --- | --- |
| Maternal early-pregnancy glycemic index (SDS) |  |  |  |  |
|  | **Mid-pregnancy** | | | |
|  | *n=2751* | *n=2754* | *n=2750* | *n=2738* |
| Basic model^b^ | -0.02 (-0.06, 0.02) | 0.01 (-0.05, 0.02) | 0.02 (-0.02, 0.06) | 0.00 (-0.04, 0.04) |
| Adjusted model^c^ | 0.00 (-0.04, 0.04) | 0.00 (-0.04, 0.04) | 0.02 (-0.02; 0.06) | 0.01 (-0.03, 0.05) |
|  | **Late-pregnancy** | | | |
|  | *n=2748* | *n=2771* | *n=2781* | *n=2769* |
| Basic model^b^ | -0.03 (-0.06, 0.01) | 0.01 (-0.03, 0.05) | -0.02 (-0.06, 0.02) | 0.00 (-0.04, 0.04) |
| Adjusted model^c^ | 0.00 (-0.04, 0.03) | 0.03 (-0.01, 0.07) | -0.01 (-0.04, 0.03) | 0.02 (-0.02, 0.06) |
|  | **Birth** | | | |
|  | *n=1575* |  | *n=1903* | *n=2824* |
| Basic model^b^ | -0.02 (-0.07, 0.04) | n.a. | -0.03 (-0.08, 0.03) | 0.00 (-0.04; 0.04) |
| Adjusted model^c^ | -0.01 (-0.07, 0.05) | n.a. | 0.00 (-0.06, 0.05) | 0.00 (-0.04, 0.04) |
|  | **Difference in head circumference SDS (95% CI)** | **Difference in abdominal circumference SDS (95% CI)** | **Difference in length**  **SDS (95% CI)** | **Difference in weight**  **SDS (95% CI)** |
| Maternal early-pregnancy glycemic load (SDS) |  | | | |
|  | **Mid-pregnancy** | | | |
|  | *n=2751* | *n=2754* | *n=2750* | *n=2738* |
| Basic model^b^ | 0.01 (-0.03, 0.05) | 0.01 (-0.02, 0.05) | 0.01 (-0.03, 0.05) | 0.02 (-0.02, 0.05) |
| Adjusted model^c^ | 0.02 (-0.06, 0.10) | 0.03 (-0.05, 0.10) | 0.07 (-0.01, 0.14) | 0.06 (-0.01, 0.14) |
|  | **Late-pregnancy** | | | |
|  | *n=2748* | *n=2771* | *n=2781* | *n=2769* |
| Basic model^b^ | 0.00 (-0.04, 0.03) | 0.03 (0.01, 0.06)* | 0.00 (-0.04, 0.04) | 0.02 (-0.02, 0.06) |
| Adjusted model^c^ | 0.00 (-0.07, 0.07) | 0.10 (0.03, 0.18)* | 0.02 (-0.05, 0.09) | 0.10 (0.02, 0.17)* |
|  | **Birth** | | | |
|  | *n=1575* |  | *n=1903* | *n=2824* |
| Basic model^b^ | 0.02 (-0.04; 0.07) | n.a. | 0.00 (-0.05, 0.06) | 0.01 (-0.03, 0.05) |
| Adjusted model^c^ | 0.07 (-0.05, 0.18) | n.a. | -0.01 (-0.11, 0.10) | 0.02 (-0.05, 0.10) |

n.a.: not available

*P<0.05

^a^Values represent regression coefficients (95% confidence interval) from linear regression models that reflect differences in standard deviation score of fetal growth and birth characteristics per one increase in standard deviation of maternal dietary glycemic index and load intake during early-pregnancy

^b^Basic models were adjusted for gestational age at study enrolment

^c^Adjusted models were the basic models additionally adjusted for maternal age, parity, educational level, prepregnancy BMI, early-pregnancy total daily energy intake, smoking during pregnancy, alcohol use during pregnancy, daily nausea and vomiting during early-pregnancy and fetal sex
